# On a Powerful and Convenient Apparatus for Locally Applying Steam

**Published:** 1814-07

**Authors:** John Spencer Harrison

**Affiliations:** Surgeon, Alstonefield, Staffordshire


*For the Medical and Physical Journal.*

### *On a powerful and convenient Apparatus for locally applying Steam; by* John Spencer Harrison, Esq. Surgeon, Alstonefield, Staffordshire.

IT is a lamentable truth, that medical gentlemen attending the bed-side of patients, are restricted from adopting many highly-valuable applications: some, though cheap, are not sufficiently convenient to be had; others require too much trouble for the practitioner to regulate and apply; and and others again are too expensive for the patient. The present communication is intended to remove one inconvenience of the first class.

None can doubt the great utility of applying the vapour arising from water, &c. in many serious diseases; but from the best apparatus being expensive, and often at a distance, it is applied in so inert and slovenly a manner (if used at all), that the patient, so far from being benefited, is commonly made worse, in consequence of the exposure occasioned by it.

What I am going to offer will show that objects near us, and which are familiar to us, seldom engross our meditation; and hence, while the slothful practitioner contents himself with administering internally, another, by an active spirit of inquiry, will make even the most common object a valuable instrument for the cure of disease.

Even the poorest house is commonly provided with a tea-kettle, and to convert this into a most powerful apparatus, the practitioner needs only to bring with him a small brass pipe, from half a yard to a yard in length. A bit of rag being now wrapped round one end of this, it may be thrust into the spout of the kettle. Care must be taken that the water in the kettle does not reach the spout, and that the lid or cover be well secured with a bit of rag and paste applied round it.

The kettle now being set upon a brisk fire, and the pipe elevated some degrees above the horizontal line, the steam will rush out in so powerful a volume as to be totally insupportable at many inches distance from the end of the pipe.

Avoiding all controversy, I shall only observe, that in cases where heat combined with moisture is adviseable, as well in external affections, as when inhaled, perhaps this may prove the most effectual way of applying it. When used generally to the head and neck, all subsequent injury may be prevented, by wiping the parts dry with a warm cloth, and applying warm flannels afterwards.

J. S. HARRISON.

